# The Effects of Routinization on Radical and Incremental Creativity: The Mediating Role of Mental Workloads

**DOI:** 10.3390/ijerph20043160

**Published:** 2023-02-10

**Authors:** Heesun Chae, Jisung Park

**Affiliations:** 1 College of Business Administration, Pukyong National University, 45 Yongso-ro, Nam-gu, Busan 48513, Republic of Korea; 2 School of Business, Chungnam National University, 99 Daehak-ro, Yuseong-gu, Daejeon 34134, Republic of Korea

**Keywords:** routinization, radical creativity, incremental creativity, mental workloads, mental effort load, time load, psychological stress load, longitudinal study

## Abstract

An important question within the creativity literature is whether routinization inhibits individuals’ creative performance. Scholars have concentrated on complex and demanding jobs that promote creativity while ignoring the potential effects of routinized activities on creativity. Moreover, little is known about the impact of routinization on creativity, and the few studies investigating this matter have reported inconclusive and inconsistent results. This study investigates the mixed impacts of routinization on creativity by examining whether routinization has a direct impact on two dimensions of creativity or an indirect impact through the mediating role of mental workloads, such as mental effort load, time load, and psychological stress load. Based on multisource and time-lagged data from 213 employee–supervisor dyads, we found a positive direct effect of routinization on incremental creativity. In addition, routinization had both an indirect effect on radical creativity via time load and on incremental creativity via mental effort load. Implications for theory and practice are discussed.

## 1. Introduction

For a long time, complex tasks have been perceived as having a positive effect on creative performance by increasing intrinsic motivation through considerable decision-making freedom and opportunities to use high-level skills and knowledge [1,2,3,4,5]. However, management researchers and practitioners continue to disapprove of the lack of creative performance by employees when performing complicated and challenging tasks [6]. One reason for the lack of creative performance is increased workload, and researchers are reluctant to view job complexity as a predictor of creativity [7,8]. Instead, very high job demands are regarded as undermining creativity: When faced with excessive workloads, employees may not have enough time and energy to anticipate, plan for, and execute changes [9]. However, interest in routinization approaches has recently increased regarding the characteristics of task design stressors that can encourage creativity rather than hinder it [10,11,12].

Often, the behaviors performed in an organization are routine responses to situations that are usually familiar, and some tasks do not require much time or energy. Such behaviors can be applied to complex tasks, regardless of their complexity. Moreover, some activities are executed repeatedly, and these tasks can be expected to yield the same results [13]. These tasks can be performed without much cognitive effort or attention, allowing the worker to think about other meaningful activities [11,12,14]. This characteristic of reducing workload through routinization is very closely related to creativity. Since creativity embodies cognitive problem solving and idea generation, it requires cognitive resources [15].

Based on this assumption, the present study addresses three related issues. By defining the different characteristics of routinization and their effects on creativity, this study examines whether routinization has a direct or indirect effect on creativity. Previous studies on the relationship between routinization and creativity are rare and inconsistent. For instance, some studies found a negative relationship, arguing that repetitive tasks increase boredom and reduce intrinsic motivation [5,16], whereas other studies revealed a positive relationship, claiming that repetitive tasks can free employees’ cognitive resources, allowing them to think about other aspects of their work [11,12,13,14]. Such inconsistent findings need clarification and call for an exploration of potential mediators that determine the relationship between routinization and creativity. Thus, in this work, we explore the mechanisms through which routinization perceived by employees affects their creative performance. Cognitive processes are identified based on which characteristics of routinization might reduce workloads, thereby increasing workers’ available cognitive potential, which they may utilize to achieve creative outcomes.

In addition, this study examines the effect of routinization through the influence of workloads on various types of creativity, such as radical creativity and incremental creativity. Creativity is broadly defined as a single-dimensional constituent concept denoting the generation of new and useful ideas [13]. However, this approach has recently been criticized for producing mixed empirical results [17,18,19]. Every job requires creative performance, for various purposes, ranging from minor adaptations or changes in how a task is performed to the production of new products or work process changes that lead to fundamental breakthroughs [20]. Accordingly, researchers have begun to emphasize the importance of creativity in various dimensions of work performance. Specifically, radical creativity is associated with dynamic changes that are substantially different from the current practices of an organization, whereas incremental creativity is associated with minor modifications to existing processes and products that do not make substantial differences to the existing framework [21]. The present study investigates the effects of routinization on both types of creativity by applying the multidimensional concept of creativity.

In short, we contribute to the current understanding of routinization and its psychological mechanisms by reconciling some of the previous mixed findings regarding creative outcomes. We explicitly show the different characteristics of routinization and their effects and then identify how these characteristics relate to creativity through the mediating role of psychological mechanisms. Specifically, we explain how routinization fosters creativity by influencing different types of mental workloads among employees, including mental effort, time, and psychological stress load. Furthermore, given the complex and multiplicative nature of creativity, we distinguish radical creativity from incremental creativity and consider the different workload drivers of each type of creativity. Finally, we empirically validate these relationships using time-lagged field data from 213 independent employee–supervisor dyads representing different industries. Ultimately, our goal is to inform theory and practice regarding the benefits of the increased employee creativity that emerges when individuals perform routinized tasks.

## 2. Theoretical Background and Hypotheses

### 2.1. Routinization and Creativity

The concept of routine is likely being harmed by its prominence: As this term becomes more widely used, its meaning becomes increasingly vague and undergoes arbitrary extensions [22]. This ambiguity concerning the many different definitions of “routine” may have contributed to the inconsistent results obtained in previous studies on the relationship between routinization and creativity. Thus, identifying researchers’ different definitions of “routinization” is a potential starting point to address before proceeding further in the conceptual development of the relationship between routinization and creativity.

The notion of “patterns” has been central to the concept of routines since the concept was introduced to clarify its inherent regularity [23]. Sidney and Winter defined a routine as a “pattern of behavior that is followed repeatedly, but is subject to change if conditions change.” [24] According to this definition, organizational routines are the antithesis of flexibility and change, as they lock organizations into inflexible and unchanging patterns of action [25]. Routine tasks oppose task complexity, which is defined as “challenging and complex jobs characterized by high levels of five core characteristics, namely skill variety, task identity, task significance, autonomy, and feedback.” [26] In other words, complex tasks, unlike routine and simple ones, allow employees to utilize their skills through a sense of control or accomplishment, which further motivates them to enhance their creativity [1,2,3,4,5]. Meanwhile, repetitive tasks lead to a narrow range of behaviors performed in familiar settings, adherence to rules and disciplinary boundaries, and the use of logic [5,16]. Therefore, routinization hinders creativity in organizations.

Mindlessness is another issue related to the characteristics of routinization that has recently attracted attention [22,23]. Several authors have emphasized that routines significantly reduce individual-level cognitive demands [11,13]. When performing routinized tasks, workers do not draw from their substantial cognitive resources from the realm of consciousness [22]. Since cognitive resources are limited and individuals cannot attend to all of their goals simultaneously, attention should be allocated selectively. Routines economize the limited information-processing and decision-making capacity of employees [27]. Meanwhile, mindfulness and complex tasks require not only attentiveness but also the conservation of attention, which could inhibit creative outcomes.

By adopting the mindlessness concept of routinization, Ohly [13] described routinization as “the automaticity in behavior.” Important features of automaticity are “unintentionality, uncontrollability, lack of awareness and efficiency” [27]. Such automatic processes occur without intention, awareness, or interference with other ongoing mental activities. Routinization is carried out by limited exploration and attention toward restricted points of the work environment. Therefore, it does not involve explicit attention to alternatives actions; thus, it involves automized behavior. It also implies that thinking procedures can occur without requiring a finite attentional system. Routinization occurs outside one’s awareness because the actor does not recognize the beginning of the process when they perform a task. Routinization also implies effortlessness in that actions are carried out without consuming any cognitive resources [27]. When the components of skill acquisition are automated through exercises, intention and attention shift from performing automated tasks to achieving higher-level aspects concerning the integration of skills [28]. Thus, routinization should not necessarily be defined as a lack of autonomy in simple work tasks or decision making.

Given that routinization, as an automatic behavior, requires little mental effort, it is expected to be positively correlated with creativity. This is because it preserves an individual’s cognitive potential, which they can subsequently use to develop new ideas. There may be an optimum level of effort that can automatically be devoted to tasks without a conscious effort.

However, few studies have adequately tested this assumption by examining the relationship between routinization and creativity [29,30]. Furthermore, the expected influences of routinization on creativity have been observed in some studies but not in others [31]. Moreover, formal specification is still lacking regarding how routinization may foster creative behaviors. Little is also known about the mediated link between cognitive mechanisms through which routines allow individuals to reduce workloads and, thus, preserve their limited information-processing and decision-making capacity. Chae and Choi recently examined the positive routinization–creativity relationship through cognitive mechanisms such as free cognitive resources [11]. However, they did not reveal which specific aspects of resources help create new and useful ideas. Further, considering that the types of creativity were not diversified or embodied, the effect of routinization could be examined in more detail by diversifying the dimension of creativity.

In short, the mixed results of previous studies on the function of potential mediators can be clarified by examining why routine tasks reduce three types of mental workloads and what specific workloads might affect two different dimensions of creativity.

### 2.2. Routinization and Mental Workload

The term “mental workload” is often defined as “the cost incurred by an individual, given their capacities, while achieving a particular level of performance on a task with specific demands.” [32] It is characterized by the demand induced by tasks requiring limited mental resources and can be considered a single or multidimensional concept [33]. The multidimensional concept of workload assumes that people can numerically express their perceived mental burdens [34,35,36].

This paper adapts Reid and Nygren’s [37] subjective workload assessment technique (SWAT) to clarify different dimensions of workloads, such as mental effort load, time load, and psychological stress load. When tasks are very complex and require significant conscious mental effort or concentration, employees might experience “mental effort load”. “Time load” refers to how often employees have spare time when conducting their daily work. When employees have no spare time, interruptions or overlaps between activities are very frequent or constant. “Psychological stress load” can be increased by confusion, risk, frustration, or anxiety, and requires determination and self-control from employees.

Routines can occur only through extensive practice and experience, which are common in many skill-acquisition situations. As familiarity with skills or tasks is gained, less attention and time need to be devoted to processes. Gradually, tasks become more automated and require only minimal resources, thus freeing the worker’s psychological resources by reducing the time cost of choosing between possible alternatives [38]. Routinization helps employees correctly use the information necessary for decision making and reminds them of critical steps or interdependent relationships when performing complex tasks [39]. As such, routinization ensures that employees perform tasks properly.

Since resources are limited and because humans have limited mental capacities, time-sharing and allocating attention may be required for all tasks. Individuals should selectively decide where to direct their attention [40]. According to theories of human information processing [40,41], when components related to automated processing are developed, the dependence on resources when performing tasks decreases, as does the sensitivity to resources [41]. Similar principles apply to the context of skill acquisition. Even resource-dependent tasks tend to progress at first and become progressively less sensitive to resources once the appropriate skills are acquired [39]. Consistent task procedures and practices are associated with high performance and reduced attention requirements. Ultimately, as tasks become more automated, quicker, and effortless, employees save time and mental energy, which reduces psychological stress.

### 2.3. Mental Workload and Creativity

Prior research indicates that creativity is a complicated construct [17,18]. Radical creativity is an intrinsic process through which a person explores novel approaches to substantially depart from existing practices. It is related to set-breaking frameworks or processes; thus, it is driven by the propensity to take risks [21,42,43]. Meanwhile, incremental creativity is an extrinsic process through which one seeks answers to current issues that need to be resolved tangibly. As such, it is driven by the need for minor modifications to adapt and conform to existing frameworks rather than an internal drive [17,42]. These creative behaviors require substantial investments of intense mental energy [44,45].

Investigations into cognitive busyness and cognitive load theories suggest that reduced cognitive capacity and overwhelmed cognitive load can restrict employees’ thinking [45] and weaken their abilities to develop novel solutions [46]. To cope with problematic situations in new ways, people need to operate outside their preferred methods; however, doing so requires substantial effort and time.

Reduced mental workloads enable employees not only to solve problems while their attention is focused elsewhere, but also to generate new ideas to improve products, processes, and procedures [11,47]. Some studies have examined the effects of high workload pressures on creativity. For example, Andrews and Smith [48] found a negative relationship between time pressure and creative performance among marketing professionals. Similarly, Amabile et al. [49] showed that employees who experienced frequent work disruptions, extreme workloads, and time pressures were almost half as creative as employees without such experiences. Furthermore, Perlow [50] studied product development engineers for four years and found that they experienced high levels of stress and low levels of creative performance when faced with high time pressures and frequent interruptions. Interestingly, however, Yu and Wang [51] revealed the opposite trend in a longitudinal study, reporting that the stressors of time pressure can increase people’s creative performance over time. Ohly and Fritz [52] also found a positive relationship between stressors and creativity.

However, no study has examined what kinds of mental workloads impact creativity. Even though adequate supplies of such mental resources are critical to creativity, these mental resources have not been classified clearly. Therefore, we investigate reduced mental workloads as a crucial resource for employees to generate radical and incremental creative outcomes. We study different aspects of mental workload because each aspect forms distinct resources triggered via routinization related to radical and incremental creativity.

### 2.4. Mediating Role of Mental Workload

The present study investigates whether routinization directly impacts two dimensions of creativity or reduces mental workloads, thus allowing employees to invest more effort in modifying existing work processes to suit current needs or suggesting radically new ways of completing processes by saving time and mental effort and increasing psychological freedom. As such, direct and indirect (positive) effects of routinization on creativity are hypothesized. By confirming these effects, this study aims to provide a better understanding of the relationship between routinization and creativity. The specific hypotheses proposed in this study are as follows:

**Hypothesis** **1.** *Routinization will be positively related to (a) radical creativity and (b) incremental creativity*.

**Hypothesis** **2.** *The direct relationships between routinization and (a) radical creativity and (b) incremental creativity will be mediated by mental workload*.

**Hypothesis** **2-1.** *The direct relationships between routinization and (a) radical creativity and (b) incremental creativity will be mediated by mental effort load*.

**Hypothesis** **2-2.** *The direct relationships between routinization and (a) radical creativity and (b) incremental creativity will be mediated by time load*.

**Hypothesis** **2-3.** *The direct relationships between routinization and (a) radical creativity and (b) incremental creativity will be mediated by psychological stress load*.

## 3. Methodology

### 3.1. Data Collection and Samples

The data used to verify the hypotheses were collected from Korean companies representing various industries, including telecommunications, electronics and manufacturing. Common method bias [53] was reduced using a survey design with two separate measuring times and sources of responses. Employees rated predictor variables, and three weeks later, their immediate supervisors rated these employees’ creativity. The researchers contacted the managers of HRD (human resources department) of the companies, and, with their permission, visited the companies that responded to the request. They then explained the purpose and response method of the survey before conducting it. The questionnaires were initially distributed to 250 employees. Three weeks after the employee survey was conducted, the outcome variable (creativity) was measured by the participants’ immediate supervisors. A total of 230 employee–supervisor dyads were collected. After eliminating questionnaires with unmatched dyads, 213 employee–supervisor dyads remained and were used for the final analysis (final response rate: 85.2%).

Among the responding employees, 66.7% were males. Age was measured according to age group to protect respondents’ personal information. The largest age group (36.2%) comprised employees in their 30s, followed by 25.4% in their 20s and 19.2% in their 40s. Moreover, 55.4% of participants held a bachelor’s degree or higher, and 28.2% had finished a two-year college program. The distribution of organizational tenure was 55.9% for less than five years, 31.1% for 5–10 years, 6.5% for 10–15 years, and 6.5% for more than 15 years.

### 3.2. Measures

Responses for all items, except those related to demographic data, were given on a seven-point scale, with options ranging from 1 (not at all) to 7 (extremely).

Routinization was assessed with the measurement technique used by Verplanken and Orbell [54]. This method measures habit strength by dividing it into specific characteristics of habit (i.e., history of repetition, automaticity and expression of one’s identity). Only five items of history of repetition and automaticity (lack of control, lack of awareness, and efficiency) which reflected behavioral characteristics were considered in this study (α = 0.75). Another aspect, namely habit (which is an expression of one’s identity), was excluded because it reflects a sense of personal style. A sample item is “I do my main tasks without consciously remembering the method”.

Workload was measured using the SWAT (subjective workload assessment technique) developed by Reid and Nygren [37]. This standardized multidimensional subjective mental workload assessment technique asks participants to judge the cognitive effort required to complete tasks. This measure comprises three scales, and each dimension (mental effort load, time load, and psychological stress load) is represented by a single item.

Radical and incremental creativity were measured using six items developed by Madjar et al. [21]. Immediate supervisors rated focal employees’ radical creativity (three items, α = 0.89; e.g., “This employee demonstrates originality in his/her work”) and incremental creativity (three items, α = 0.83; e.g., “This employee uses previously existing ideas or works in an appropriate new way”).

Previous research [18,42] controlled four demographic variables (i.e., employees’ age, gender, organizational tenure, and education level), which were confirmed to be significantly related to creativity. In the present study, gender was measured as a dichotomous variable (women = 0; men = 1) and organizational tenure was measured in years. Age was coded according to age group. Participants aged under 20 years were coded as 1, while those in their 20s, 30s, 40s, and 50s were coded as 2, 3, 4, and 5. Finally, education level ranged from high school graduation to master’s degree or higher (high school graduation = 1; two-year college graduation = 2; undergraduate degree = 3; and master’s degree or higher = 4). All hypothesis test results reported below were identical, independent of these control variables [55].

### 3.3. Analytical Strategy

To test the hypothesized relationships between latent and measured variables, we used a multivariate analysis technique with structural equation modeling (SEM) by employing AMOS 22 software. This is an efficient analysis technique for simultaneously examining multiple dependence relationships between latent variables. Following the work of Anderson and Gerbing [56], we verified the measurement model and the structural model in separate steps. In the first measurement model stage, we analyzed the discriminate validity of the proposed constituent concepts through confirmatory factor analysis. In the second structural model stage, we evaluated several structural models to analyze the hypothesized structural relationship between the latent and measured variables.

## 4. Results

### 4.1. Descriptive Statisticss

The means, standard deviations, and correlations of the variables included in the present study are reported in Table 1. Education level (r = 0.29, *p* < 0.001) was positively correlated with radical creativity, but mental effort load (r = −0.16, *p* < 0.05) and time load (r = −0.19, *p* < 0.01) were negatively correlated with radical creativity. Incremental creativity was positively related to education level (r = 0.18, *p* < 0.05) and routinization (r = 0.15, *p* < 0.05), but negatively related to mental effort load (r = −0.22, *p* < 0.01). In addition, routinization had a negative correlation with mental effort load (r = −0.16, *p* < 0.05) and time load (r = −0.22, *p* < 0.01).

### 4.2. Measurement Model

We analyzed the measurement model before testing the structural model using confirmatory factor analysis without including control variables. We performed confirmatory factor analysis to compare additional explainable models. These included (1) the hypothesized six-factor model, which represents the measurement model of this study; (2) a five-factor model combining mental effort resource and time resource; (3) a four-factor model combining mental effort resource, time resource, and psychological resource; (4) a three-factor model combining routinization and three dimensions of work resources; (5) a two-factor model combining radical and incremental creativity; and (6) a single-factor model. The results of the hypothesized six-factor model indicated a good fit with the data, χ²[65] = 169.1, *p* < 0.001; CFI = 0.91, TLI = 0.90, RMSEA = 0.08 (Table 2). This fit was better than that achieved by any of the alternative models. All factor loadings on the specified factor were significant at the 0.001 level. These results justified the further analysis conducted in the next step (structural model).

### 4.3. Structural Model

We used several models including control variables to investigate whether routinization influences two types of creative performance directly or whether workload processes mediate these influences. First, we modeled the proposed direct relationship (direct-effect-only model). The coefficient for the direct path to radical creativity was not significant (β = 0.15, ns.), but the direct path to incremental creativity was significant (β = 0.17, *p* < 0.05). The global fit indexes were not particularly good, χ²[82] = 471.0 *p* < 0.001; CFI = 0.69, GFI = 0.68, RMSEA = 0.15 (Table 3). These findings provide empirical support for Hypothesis 1(b), but not Hypothesis 1(a).

**Figure 1:**
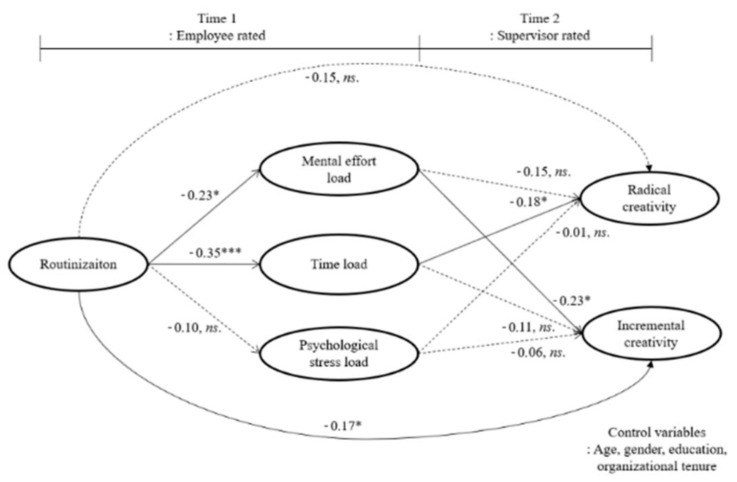
The hypothesized model for routinization and creativity with structural model parameter estimates. Thicker lines represent statistically significant results. Dotted lines represent statistically nonsignificant results. *ns* = not statistically significant, *** < 0.001, * < 0.05.

Second, we tested an indirect model in which three different workloads cooperated to mediate the relationship between routinization and radical and incremental creativity (hypothesized-mediated model). Figure 1 illustrated the indirect structural model with the derived path coefficients. This model showed good fit with the present data, χ²[117] = 331.8, *p* < 0.001; CFI = 0.90, GFI = 0.90 RMSEA = 0.09, and is a significant improvement over the direct model, Δχ² [35] = 39.2, *p* < 0.001 (Table 3). Specifically, we found a negative relationship between routinization and mental effort load (β = −0.23, *p* < 0.05); in turn, mental effort load was negatively related to incremental creativity (β = −0.23, *p* < 0.05). Furthermore, routinization was negatively related to time load ( β= −0.35, *p* < 0.001), and time load was negatively related to radical creativity (β = −0.18, *p* < 0.05).

Third, we assessed an alternative structural model by adding the direct path between routinization and radical and incremental creativity (alternative model-including direct path). As shown in the fourth row of Table 3, although this alternative mediation model including a direct path fits the data well, χ²[115] = 327.1, *p* < 0.001; CFI = 0.90, GFI = 0.90 RMSEA = 0.09, the chi-squared change of the alternative model was not statistically better than that of the initial mediating estimation model, χ²[2] = 4.7, ns. This finding supports the parsimonious initial mediation model over the alternative model-including direct path.

Moreover, following the work of Preacher and Hayes [57], we conducted bootstrapping analysis to assess the indirect effects of mental workloads on the relationship between routinization and radical and incremental creativity. We performed 1000 bootstrap replicates and found that time load significantly mediated the relationship between routinization and radical creativity (indirect effect = 0.05, 95% bias-corrected CI [0.01, 0.11] (Table 4)). Thus, Hypothesis 2-2(a) is supported. Moreover, mental effort load significantly mediated the relationship between routinization and incremental creativity (indirect effect = 0.04, 95% bias-corrected CI [0.00, 0.11]), supporting Hypothesis 2-1(b).

## 5. Discussion

### 5.1. Overall Findings

The main purpose of this study is to explore the potential positive effect of routinization on different types of creativity by identifying the mediating role of mental workloads. The analysis included multisource and time-lagged data consisting of 213 employee–supervisor dyads. The results of structural equation modeling and bootstrapping analysis verified that routinization exerted a significant positive effect on incremental creativity but its direct effect on radical creativity was not significant. Thus, these findings only support hypothesis 1b. The results provide further empirical evidence that routinization has an indirect beneficial effect on both studied types of creativity through the mediating role of mental workloads. Specifically, routinization exhibited a significant indirect effect on radical creativity via time load and a significant indirect effect on incremental creativity via mental effort load. These outcomes support Hypotheses 2-1(b) and 2-2(a). The present analysis provides significant implications for research and practice as discussed below. This section also specifies the limitations of this study and provides directions for further studies.

### 5.2. Theoretical Implications

This study contributes to the literature on routinization and creativity in several meaningful ways. This study’s most important contribution is related to its reexamination of the impact of routinization on creative performance. Routinization is not expected to generate direct rewards because it is not traditionally regarded as a factor that fosters creativity [22,23]. Unfortunately, the importance of routinization has been overlooked since it has been related to tedious and simple repetitive tasks; thus, it is considered the opposite of job complexity [11,12,13]. However, the recent results of warning studies indicate that challenging and complex tasks can hinder creativity by causing stress at work and strains such as cognitive overload [1,2,3,4,5,58,59]. These issues have increased the interest in routinization among researchers [9,11,12,13]. In light of this recent knowledge, the current study developed a theoretical framework for assessing the positive relationship between routinization and creativity.

First, we recognized the different definitions of “routinization” provided by researchers. We also found that previous studies on the relationship between routinization and creativity are rare and provide inconsistent results. Most studies have reported a negative relationship, arguing that, unlike challenging and complex tasks, repetitive tasks increase boredom and reduce intrinsic motivation [5,16]. Recent studies have emphasized room for thought and incubation (as opposed to excessively challenging tasks or pressure) as critical to the expression of creativity [14,25]. Moreover, mindlessness, a characteristic of another dimension of routinization, was highlighted [11,13]. Few studies have clarified the positive relationship between routinization and creativity through which the mindlessness generated by routine tasks frees employees’ cognitive resources, allowing them to think about other aspects of their work.

The present study extends this discussion by exploring the positive aspects of routinization, which is defined as the automaticity of performing tasks that result in reduced mental workloads. Routinization is often regarded as a source of stability or inertia due to performing repetitive tasks over time [55]. However, according to the theory of human information processing [40], routinization is an automized behavior that does not require explicit consideration of alternative courses of action [39]. It requires constrained attention and the exploration of restricted aspects of the environment. The more automated tasks become, the less conscious processing and attention they require. Thus, we assessed the relationship between routinization and creativity by emphasizing the importance of routines. We found that the more task behavior an employee can store in their subconscious, the greater their capacity for conscious processing is.

Second, through a reconceptualization of creativity as radical and incremental [17,18], this study presented a possible way to resolve the mixed findings about the existing relationship between routinization and creativity. The results of this study revealed that routinization predicted incremental creativity but not radical creativity. Both radical and incremental creativity help people solve problems and perform tasks well. However, radical creativity is related to paradigm-breaking or revolutionary work, whereas incremental creativity is related to minor improvements and adaptations [21,42,44]. Automized behaviors may filter out aspects of a situation that suggest change and novel approaches while increasing the salience of aspects that focus on established patterns. This pattern leads employees to establish standards and skews the interpretation of a situation toward automized patterns. The current findings align with the results of Madjar et al. [21], who provided evidence for this view by finding that conformity and standardization facilitate small modifications and incremental changes.

Third, as an extension of Chae and Choi’s research [11], this study has great theoretical significance by revealing which specific mental workloads are minimized by routinization, thus leaving workers with adequate mental resources to be creative. As cognitive busyness and cognitive load theories [45] emphasize, creative work requires a substantial and continuous stream of mental resources. By classifying mental workloads into three dimensions, this study revealed that routinization can reduce mental effort load and time load but not psychological stress load. Creativity is a complex phenomenon that can have multiple influences and requires a substantial amount of effort [15]. People must operate outside their preferred methods to cope with problems in creative ways; however, doing so comes at the expense of more effort and time [18]. This study revealed that, instead of reduced confusion, frustration, or anxiety, time and mental effort are needed to increase creative performance [60]. This outcome is significant because previous studies dealt with mental resources in a fragmented way [49,50,51] by dividing them into three dimensions and examining the different effects in detail.

Fourth, we examined the possible emergence of different creativity types from the same task characteristic (routinization), which can be considered when instigating different types of mental workload among employees. The results showed that radical creativity can be promoted when employees’ time load is reduced through routinized tasks. Meanwhile, incremental creativity can be promoted when employees’ mental effort load is reduced through such tasks. One explanation for these results may be related to the type of creativity and types of resources (specific vs. general for creativity) considered. Resources are needed to enable creativity [14], and this study focuses on the mental effort and time resources that specifically facilitate different types of creativity. Most incremental ideas require few resources and may benefit little from additional time and support [18]. However, since radical ideas involve more risks and typically require various applications of knowledge and multiple challenging attempts, they substantially depart from existing practices [17]. Therefore, additional time-consuming flexibility is needed to buffer these risks and increase the possibility of generating radical breakthroughs.

Previous findings about time-related variables related to creativity have reported contradictory results, including negative [61,62], positive [51,52], nonlinear [13,62], and nonsignificant effects [15]. The present study contributes to resolving such inconsistencies by subdividing and reexamining different aspects of creativity. In particular, this study revealed that reduced time load fosters radical creativity. Thus, this study revealed differential antecedents on creativity types, suggesting that future studies should examine which factors promote different types of creativity.

Fifth, by using a time-lagged data set, we tried to gather strong support for a mediating mechanism. Specifically, we found that routinization benefits creativity through the mediating role of three dimensions of mental workload. However, the temporal implications of routinization could be examined more precisely by confirming the time difference of the mediation effect by dividing it into two periods. A longitudinal study is needed to draw firm conclusions about the causal directions between the investigated variables.

### 5.3. Practical Implications

Although predominant research considers routinization an undesirable task characteristic, our research shows that routinization leads to creative behaviors at work. The extent to which routinization potentially benefits organizational functioning largely depends on how tasks are designed. Today, employees face the pressure of generating creative ideas in highly complex and stressful working situations. Managers should remember that mental workload severely inhibits creative outcomes [8,9] since creativity requires time and effort. Tasks with low cognitive difficulty provide workers with the attention capacity necessary for creative thinking [11]. That is, the more task behavior an employee can store in their subconscious, the greater their capacity for conscious processing is, which enhances creative performance.

Individual employees should also recognize that creative performance can be promoted through routinized tasks and try to take advantage of the reduced mental workload associated with routinization to generate new ideas. Managers should also remember that they can benefit from employees’ creativity even when individuals are performing routinized tasks. Given that managers can influence how employees experience their work and recognize how work is designed, they should consider that providing challenging or demanding tasks does not always boost individuals’ creativity. Conversely, it might lead to work-related stress, resulting in burnout.

Given that radical and incremental creativity are motivated by different mental workloads [18,21], another benefit of the current results is that they provide a better understanding of the predictors of radical versus incremental ideas. Managers should specify the desired creativity type in their teams or organizations. The value of radical and incremental creativity can vary across tasks and social factors (e.g., task performance, goals, and norms). For example, radical creativity may be appropriate for employees or teams that have to solve unstructured problems in an uncertain task environment involving major changes. In contrast, incremental creativity may be appropriate for employees or teams that need to improve or modify routine procedures under relatively stable conditions.

Thus, managers may need to obtain a better understanding of the complex cognitive processes undertaken by employees. They can then use leadership strategies and other interventions to reduce employees’ mental workload, thereby focusing employees’ creative efforts in a specific direction. Our analysis indicates that managers who prefer challenging and risky forms of creativity should intentionally reduce time resources by organizing tasks involving automated behavior patterns instead of fully occupied tasks that are cognitively overloaded. In contrast, employees who experience reduced mental effort can effectively promote smaller modifications through creativity.

Furthermore, by providing the creative norms of the organization, managers can more effectively stimulate more desirable types of creativity, especially regarding ideas that will benefit the firm. For example, if managers indicate that the organization prefers creativity to lead to minor modifications, employees will be preoccupied with creating small improvements to existing ideas instead of providing unwelcome radical solutions. To this end, organizations can provide leadership training and development programs to help managers accurately identify the preferred types of creativity and analyze which resources are needed for their employees to guide creative efforts in the right direction.

### 5.4. Limitations and Future Directions

The contributions of this research should be viewed in light of several limitations. First, we did not identify situational contingencies, such as the individual and managerial situations necessary for employees’ creativity (e.g., individual dispositions, leadership styles, and climates). Consistent with the componential perspective dominant in the creativity literature, a further explanation is needed regarding how employees can be encouraged to allocate their mental workloads to generate radical and incremental creativity. Second, we measured three dimensions of mental workload using a single-item measure. Measurements of cognitive and mental workload have gained increasing credence in cognitive load theory, working memory research, and cognitive theories of multimedia learning. Cognitive load is an internal information-processing technique and can be treated as a theoretical concept that cannot be measured by direct observation [63]. However, people can provide numerical indicators of perceived mental burdens such as mental effort load, time load, and psychological stress load. Therefore, a valid and reliable tool has been developed to evaluate cognitive load using multidimensional rating scale techniques [34,35,36]. Beyond testing in experiments employing the dual-task method, researchers should examine how these theories and techniques can be expanded in practical conditions.

## 6. Conclusions

This study explores the renewed interest in routinization in the creativity literature by suggesting the potential positive effect of routinization on different types of creativity through the mediating mechanism of workloads. The present study’s results will hopefully increase other researchers’ interest in reinterpreting routinization and encourage them to take a more diverse approach to its relationship with creativity.

## Figures and Tables

**Table 1:** Means, standard deviations, and inter-scale correlation.

	Mean	S.D	1	2	3	4	5	6	7	8	9
1. Age	2.89	1.22									
2. Gender	0.33	0.47	−0.45 ***								
3. Education	2.43	0.81	0.26 **	−0.16 *							
4. Organizational tenure	5.05	4.81	0.58 ***	−0.21 **	−0.09						
5. Routinization	4.52	0.79	0.02	0.07	−0.01	0.13					
6. Mental effort load	4.51	1.09	−0.01	−0.15*	−0.02	−0.06	−0.16 *				
7. Time load	3.23	1.22	−0.24 ***	0.08	−0.22 **	−0.07	−0.22 **	−0.09			
8. Psychological stress load	4.68	1.20	−0.11	−0.01	−0.05	−0.08	−0.06	0.62 ***	−0.06		
9. Radical creativity	4.71	1.03	0.01	−0.06	0.29***	−0.09	0.11	−0.16 *	−0.19 **	−0.11	
10. Incremental creativity	4.83	0.90	−0.09	0.08	0.18*	−0.08	0.15 *	−0.22 **	−0.13	−0.08	0.65 ***

Note: *N* = 213, *** < 0.001, ** < 0.01, * < 0.05. Two-tailed.

**Table 2:** Comparison of measurement models.

Model	No. of Factors	χ²	df	Δχ²	RMSEA	CFI	TLI
Baseline model	6 factors: Rou, MEL, TL, PSL, RC, IC	169.1	65		0.08	0.91	0.90
Model 1	5 factors: Rou, (MEL +TL), PSL, RC, IC	190.6	69	21.5 *	0.09	0.89	0.85
Model 2	4 factors: Rou, (MEL + TL + PSL), RC, IC	209.4	72	40.5 *	0.10	0.87	0.84
Model 3	3 factors: (Rou + MEL + TL + PSL), RC, IC	281.6	75	112.5 **	0.11	0.81	0.77
Model 4	2 factors: (Rou + MEL + TL + PSL), (RC + IC)	358.4	76	189.3 **	0.13	0.75	0.70
Model 5	1 factor: (Rou + MEL + TL + PSL + RC + IC)	494.7	77	325.6 **	0.16	0.63	0.56

Note: ** <0.01, * <0.05; Rou = routinization; MEL = mental effort load; TL = time load; PSL = psychological stress load; RC = radical creativity; IC = incremental creativity; RMSEA = root mean square error of approximation; CFI = comparative fit index; TLI = Tucker–Lewis index.

**Table 3:** Comparison of measurement models.

Model	χ²	df	RMSEA	CFI	TLI
1. Measurement model	169.1	65	0.08	0.91	0.90
2. Direct-effect-only-model	471.0	82	0.15	0.69	0.68
3. Hypothesized mediating model	331.8	117	0.09	0.90	0.90
4. Alternative model (including direct path)	327.1	115	0.09	0.90	0.90

Note: Chi-squared values for the models are all significant at *p* < 0.001.

**Table 4:** Results of bootstraps for indirect effects.

Mediator	Dependent Variable	Effect Size	Bias-Corrected Confidence Intervals	
			Lower	Upper
Mental effort load	Radical creativity	0.03	−0.001	0.093
**Time load**		**0.05**	**0.011**	**0.113**
Psychological stress load		0.00	−0.014	0.035
**Mental effort load**	Incremental creativity	**0.04**	**0.004**	**0.111**
Time load		0.03	−0.002	0.089
Psychological stress load		0.00	−0.046	0.008

Note: The level of confidence intervals (CIs) is 95%.

## Data Availability

Data is unavailable due to privacy or ethical restrictions.
